# Socio-economic and ethnocultural influences on geographical disparities in breast cancer screening participation in Victoria, Australia

**DOI:** 10.3389/fonc.2022.980879

**Published:** 2022-11-29

**Authors:** Geoffrey W. Stuart, James A. Chamberlain, Roger L. Milne

**Affiliations:** ^1^ Cancer Epidemiology Division, Cancer Council Victoria, Melbourne, VIC, Australia; ^2^ Faculty of Medicine, Dentistry and Health Sciences, The University of Melbourne, Melbourne, VIC, Australia; ^3^ Centre for Epidemiology and Biostatistics, Melbourne School of Population and Global Health, The University of Melbourne, Melbourne, VIC, Australia; ^4^ Precision Medicine, School of Clinical Sciences at Monash Health, Monash University, Clayton, VIC, Australia

**Keywords:** breast cancer, cancer screening, socio-economic factors, ethnicity, culture, geographical areas

## Abstract

**Objective:**

To examine the socio-economic and ethnocultural characteristics of geographical areas that may influence variation in breast cancer screening participation.

**Methods:**

In a cross-sectional analysis breast cancer screening participation for statistical areas in Victoria, Australia (2015-2017) was linked with data from the 2016 Australian Census. We selected four commonly used area-level measures of socio-economic status from the Australian Census (i) income (ii) educational level (iii) occupational status and (iv) employment profile. To assess the ethnocultural characteristics of statistical areas we used the Census measures (i) country of birth (ii) language spoken at home (iii) fluency in English (iv) religion and (v) the proportion of immigrants in an area, together with their recency of migration.

**Results:**

All the selected measures were related to screening participation. There was a high degree of association both within and between socio-economic and ethnocultural characteristics of areas as they relate to screening. Ethnocultural characteristics alone accounted for most of the explained geographical disparity in screening participation.

**Conclusions:**

Geographical disparities in breast cancer screening participation may be due to ethnocultural factors that are confounded with socio-economic factors.

## Introduction

Breast cancer is the most common cancer in women (1) and has one of the best survival rates of all cancers in countries with advanced health care systems, particularly if it is diagnosed early (2). There is broad consensus that regular mammography screening lowers breast cancer mortality in women aged 50-69 years (3–5). If breast cancer is diagnosed at a later stage, not only is mortality greater, but treatment costs are higher (6). Australia has a well-established free biennial breast screening program, with 55% of women aged 50-74 years invited to present for screening attending (7). This screening participation varies by geographic area, as does the socioeconomic and ethnocultural characteristics of the residents.

While the existence of socioeconomic (8) and ethnocultural (9, 10) disparities in breast screening participation has been established by previous Australian research, the evidence for these interdependent factors influencing screening participation is not entirely consistent across studies. This is illustrated by a recent analysis of socio-economic and ethnocultural correlates of breast screening participation in Australia which was limited to two broad measures (7). The first measure was of socio-economic status (SES) using the quintiles of the Australian Bureau of Statistics (ABS) index of the composite measure of relative socio-economic disadvantage (IRSD) (11). Women aged 50–74 living in the most disadvantaged socioeconomic areas had a lower age-standardised participation (52.1%) than women living in the least disadvantaged areas (54.6%). This suggests that SES has only a minimal effect on screening participation, consistent with the fact that screening is free. The second measure was an ethnocultural measure: whether a language other than English was spoken at home. Based on individual data, those who spoke English at home were much more likely to participate in screening (56.3%) compared to those who spoke another language at home (45.4%).

The relationship between ethnocultural measures and screening is supported by previous studies within particular ethnic communities which have found lower breast screening participation in Indian-Australian women (12), Chinese-Australian women (13) and Arab-Australian women (14), albeit in samples of convenience. Research using broader samples has produced inconsistent findings. Achat et al. (15) surveyed a stratified random sample of participants from the breast screening program of the Australian state of New South Wales. Although the survey was limited to those with adequate English proficiency, a language other than English being spoken at home was associated with lower screening participation. Employment status did not affect screening participation, and income was only weakly related, with significant differences associated only with the very lowest income category. Birch et al.(2007) found that higher income was associated with higher screening participation, and being born overseas with a lower screening participation, even after mutual adjustment for association (16). Weber et al. (2013) found that higher screening participation was associated with higher educational level, higher income, and being in full or part-time work, but lower participation was associated with speaking a language other than English at home or being born overseas (17). Byles et al. (2014) reported that higher educational level was associated with *lower* screening participation, but employment was not related to screening participation (18). They did not include any ethnocultural measures in their survey.

It is difficult to separate out the socioeconomic measures from the ethnocultural measures because socioeconomic and ethnocultural disparities are correlated. One reason for this is that more recently arrived migrants from some source countries, who may be less acculturated and less fluent in English, also tend to have lower levels of socio-economic advantage (19). As well, composite indices such as IRSD (11) do not capture all the information in the component measures (20) and this may partly explain some of the weak relationships observed between screening participation and SES. The somewhat inconsistent results may also reflect the complex relationships between ethnocultural and socio-economic determinants of breast screening participation. The findings of particular studies might be affected by (i) sample selection, especially if fluent English is a requirement for participation (ii) which specific measures of socio-economic and ethnocultural status are used, and (iii) how interdependence both between and within these groups of measures is treated. The relative contribution of socio-economic and ethnocultural factors to breast screening participation has important implications for the design of interventions to improve participation. To gain further insights into this issue, we analysed complete (i.e., unselected) data from the breast screening program of the state of Victoria for the years 2015-2017, linked to area-based measures of socio-economic and ethnocultural status from the 2016 Australian Census.

## Methods

This study is a cross-sectional observational study, combining the summary screening data for geographic areas from Breastscreen Victoria with the ABS census data. The unit of analysis was the geographic areas defined by the ABS (21) as statistical areas (SA2).

The sample consisted of all Victorian women in the age range 50-74 screened by BreastScreen Victoria between 1 July 2015 and 30 June 2017, including women invited to screen and women who volunteered to screen. All women registered on the electoral roll and aged 50-74 are invited to participate in screening mammography by BreastScreen Victoria. If a woman screened more than once in the time period, then only her latest screening episode was considered. Women who undertook private mammography screening were not captured in the dataset. Data were stratified into 5-year age bands. No other demographic data were provided for individuals, other than the ABS SA2 of residence (21). These areas have an average population of approximately 10,000 persons in populated areas, with less in some regional or remote areas. The denominator for calculating the percentage screening was based on ABS estimated SA2 residential population for 2016 obtained from census data, rather than the number of invitations sent out. There may be screening done from invitations sent out prior to 1 July 2015 included in the numerator. Fourteen sparsely populated or unpopulated SA2s were excluded from analysis, as were four areas near the state border, where the nearest breast screening clinic was in the neighbouring state. The remaining 444 SA2s were retained in the analysis. This represented an aggregate total of 812,522 women eligible for screening. The median population of women in the age range 50-74 per SA2 was 1,696 (5^th^ percentile: 612; 95^th^ percentile:3,302).

The ABS assign a categorical “remoteness” index to each statistical area used in the Australian census, based on the Accessibility/Remoteness Index of Australia (ARIA+). The continuous index is reduced to five categories by the ABS, namely “Major Cities”, “Inner Regional”, “Outer Regional”, “Remote” and “Very Remote” (21). The state of Victoria has no very remote areas and a very small proportion of remote areas. For analysis purposes the remote areas were merged with the outer regional areas, yielding three categories of rurality.

Age-adjusted screening participation for each SA2 was calculated by weighting the proportion of invitees attending within each age stratum according to the average number within each age stratum across the whole sample.

The ABS has developed a set of composite measures of SES, the socio-economic indices for areas (SEIFA) indices (11). These measures are widely used in area-based public health studies and were used to enable comparisons with previous research. For our study we also selected four commonly used separate area-based measures of SES from the Australian Census (i) income (ii) educational level (iii) occupational status and (iv) employment profile.

There is no analogue of the SEIFA indices for the ethnocultural domain. To quantify the ethnocultural characteristics of statistical areas we used the Census measures (i) country of birth (ii) language spoken at home (iii) fluency in English (iv) religion and (v) the proportion of immigrants in an area, together with their recency of migration.

Each of the measures above is composed of a number of categories, which are occasionally ordered, for example, income bands (see **Supplementary material** for details). Prior to the main statistical analysis, we developed summary scores representing each Census measure. We initially included categories that represented a reasonable proportion (over 1%) of residents on average across SA2s, or otherwise a greater proportion in a subset of SA2s (e.g., Jewish religious affiliation). For the purpose of characterising areas, all residents (not just women in the screening age range) were included in the analysis. In some cases, persons under 15 years of age were excluded from consideration (e.g., from occupational status, where they are coded as “not applicable”).

Following initial category selection, for each area the proportion of residents in a given category was plotted against screening participation to identify any non-linear relationships. Quadratic terms were included in models when they significantly (p<.05) contributed to univariate regression model fit. For each measure, starting with the initial set of categories (including any relevant quadratic terms), the weakest contributors in terms of semi-partial correlation were dropped from standard multiple regression models, as long as the model fit was not significantly worse as measured by a likelihood ratio test (p<.05) than the starting model. Parameters representing the spatial autocorrelation between areas were then introduced into the models, using the Stata 16 command *spregress*. In the presence of spatial autocorrelation, treating areas as independent observations violates the assumptions of standard regression, and inflates significance levels (22). Spatial correlation parameters were derived from the correlation between the residual estimates of screening participation between contiguous regions. This method was used rather than one based on spatial correlation as a function of distance. This was because statistical regions had similar populations but differed widely in area (and hence distance to the centroids of the nearest neighbours), especially between rural and urban locations. Using spatial regression, further categories were excluded from models if they did not make a significant contribution to model fit (according to a likelihood ratio test) in the presence of spatial autocorrelation. Importantly, the inclusion of a category and its weight in the composite score derived from multiple regression analysis cannot be directly interpreted as a measure of importance, because the categories are not independent at an area level. The fitted (predicted) values from the final regression models were then retained as summary scores for that measure. Full details of model construction for each measure are provided in the online **Supplementary material**.

We examined how the derived composite measures related to breast screening participation, both separately and in combination. Univariate and multiple linear regression was used to predict area-based screening participation from the nine derived socio-economic and ethnocultural measures. Interdependence within and between the two groups of measures was assessed using Pearson correlations and nested model comparisons. Relative fit was quantified with R^2^ and pseudo R^2^ indices together with semi-partial correlations and their associated significance tests. The squared semi-partial correlation gives an indication of how much of the multiple R^2^ is independent of other predictors. To provide a complementary estimate of how much the separate scores overlapped in predicting screening participation, principal components analysis was used to derive general scores (or “G-scores”) representing the variance shared by a set of measures, based on the matrix of correlations. The first principal component score was used as the G-score. These scores complement semi-partial correlations because they represent the variance that a set of measures have in common (for example, the set of measures used to quantify the ethnocultural characteristics of areas). Comparison between regression models using different combinations of socio-economic and ethnocultural characteristics, as well as rural-urban differences, was based on spatial regression, including spatial autocorrelation terms as described above. The association of screening participation with remoteness was considered last because rural areas may have both less cultural diversity and a different distribution of SES. This may partly account for the effect of remoteness on screening participation. Adding remoteness to models last therefore provided a better estimate of the independent effect of distance from screening services on screening participation.

## Results

**Table 1** shows the relationship between the socio-economic and ethnocultural measures and breast screening participation, using standard regression (i.e., ignoring the spatial nature of the data). Individually, all had a moderate relationship with screening participation. As shown in **Table 2**, all the measures were correlated with each other, sometimes quite strongly. Returning to **Table 1**, within the socio-economic domain, only occupation and education independently accounted for a proportion of variance in screening participation greater than 0.01 (sr^2^ = 0.056 and 0.054 respectively) where the multiple R^2^ for this set of measures was 0.30. Within the ethnocultural domain, only country of birth independently explained more than 0.01 of the variance in screening participation (sr^2^ = 0.023), against a multiple R^2^ of 0.34. In the combined model using all nine measures, only occupation and country of birth independently contributed more than 0.01 to overall model fit (sr^2^ = 0.015 and 0.016 respectively), where multiple R^2^ was 0.36.

**Table 1:** Relationship of individual socio-economic and ethnocultural domain scores to area-based screening participation.

Domain	Univariate Regression		Domain Specific Multiple Regression		Combined Domain Multiple Regression	
	r^2^	p	sr^2^	p	sr^2^	p
Socio-economic						
*Income*	.146	<.0001	.003	.15	.000	.83
*Occupation*	.119	<.0001	.056	<.0001	.015	.001
*Education*	.212	<.0001	.054	<.0001	.003	.18
*Employment*	.201	<.0001	.010	.02	.001	.54
Ethno-cultural						
*Country of Birth*	.303	<.0001	.023	.0001	.016	.001
*Recency of Migration*	.216	<.0001	.006	.05	.003	.13
*English Fluency*	.100	<.0001	-.003	.19	-.003	.18
*Language at Home*	.250	<.0001	.001	.54	.000	.65
*Religion*	.298	<.0001	.010	.01	.005	.07

Domain specific regression models represent multiple regressions including only the measures within each domain. The combined regression model included all nine measures. Squared semi-partial correlations (sr^2^) represent the unique contributions of each measure to screening participation after controlling for all other measures in the multiple regression model. For details of the development of each measure see online **Supplementary material**.

**Table 2:** Pairwise Pearson correlations between socio-economic and ethnocultural characteristics of areas, as related to breast screening participation.

	1	2	3	4	5	6	7	8
1.Occupation	–							
2. Employment	.35	–						
3. Education	.13	.68	–					
4. Income	.21	.71	.79	–				
5. Recency of Migration	.25	.62	.66	.61	–			
6. Country of Birth	.37	.66	.67	.50	.73	–		
7. Religion	.46	.66	.72	.62	.66	.79	–	
8. Language*	.40	.72	.80	.79	.66	.74	.88	–
9. English Fluency	.28	.56	.45	.53	.70	.58	.50	.63

*Language refers to language spoken at home and is related to but not the same as English fluency

The next step in the analysis was to further examine how these correlated measures combined to influence screening participation, and to compare their performance with the commonly used SEIFA composite indices − in particular the IRSD measure of relative socio-economic disadvantage. At this point spatial regression was introduced, and the remoteness measure was added to regression models. **Table 3** shows the overall fit of various spatial regression models, starting with one that included only IRSD. The second column of the table shows the pseudo R^2^ values associated with the fit of various models. IRSD alone accounted for only a small proportion of the variance in screening participation by area. When the three other SEIFA indices were included (relative advantage and disadvantage [IRSAD]; economic resources [IER] and education and occupation [IEO]), fit improved (Pseudo R^2^ = 0.12), but not to the level obtained using the full set of individual socio-economic measures (Pseudo R^2^ = 0.303). Using only the ethnocultural measures, fit was even better, very close to the fit of the full model that included all nine measures. This reflects the correlation between the individual measures shown in **Table 2** and the individual semi-partial correlations shown in **Table 1**. In summary, it was possible to model screening participation using only ethnocultural measures with very little improvement when socio-economic domain scores were added to the model. The level of association between the two groups of measures was high.

**Table 3:** Comparison of various spatial regression models of the relationship between socio-economic and ethnocultural characteristics of areas and breast screening participation.

Model	Pseudo R^2 *^(all p<.0001)	+ Remoteness ΔR^2^	p
Null	—	.036	<.001
IRSD	.041	.068	<.001
All 4 SEIFA measures	.120	.028	<.001
All 4 socio-economic measures	.303	.009	.03
Socio-economic G-score	.254 (83.9%)	.012	.01
All 5 ethno-cultural measures	.342	.000	.48
Ethnocultural G-score	.306 (89.5%)	.000	.87
All 9 measures	.363	.002	.20
All measures G-score	.311 (86.7%)	.000	.31

IRSD, index of relative socio-economic disadvantage.

SEIFA, socio-economic indices for areas.

G-Score: First principal component of the correlation matrix of individual measures within a model Percentages associated with G-scores (in brackets) indicate the relative fit of univariate spatial regressions using G-scores, compared to the fit of multivariate models using the corresponding set of individual measures (row above).

Remoteness ΔR^2^ represents the improvement in pseudo R^2^ when the remoteness index of areas was added to each model.

*There is no direct equivalent to the R^2^ value obtained from ordinary least squares multiple regression in spatial regression. Model fit is given by the correlation between the observed screening values and the predicted values from the spatial regression model (including spatial autocorrelation terms and fitted using iterative procedures).

When screening participation was predicted from G-scores using spatial regression, the pseudo R^2^ was consistently greater than 80% of that of a multiple spatial regression model using the corresponding set of individual measures (**Table 3**). Given that this was true of the G-score representing the combination of both socio-economic and ethnocultural measures, there is a considerable degree of redundancy between the individual measures in characterising statistical areas in relation to screening participation. For the purpose of predicting area-based screening participation, ethnocultural measures on their own were largely sufficient.

**Table 3** also shows the improvement in the fit of spatial regression models when the ABS remoteness index was added. Remoteness only had an independent effect on screening in addition to socio-economic measures but not ethnocultural measures. This was strongest for SEIFA indices, but this may indicate that the remoteness variable captured socio-economic variation between areas missed by those composite indices. Remoteness accounted for more variance in screening participation when combined with the SEIFA IRSD index than it did when modelled on its own, representing a suppressor effect. When all four individual socio-economic measures were included in the spatial regression model, remoteness made a slight and marginally significant contribution to model fit. When the ethnocultural measures were in the model, alone or in combination with socio-economic measures, remoteness made only trivial and non-significant contributions to model fit.

**Figure 1:**
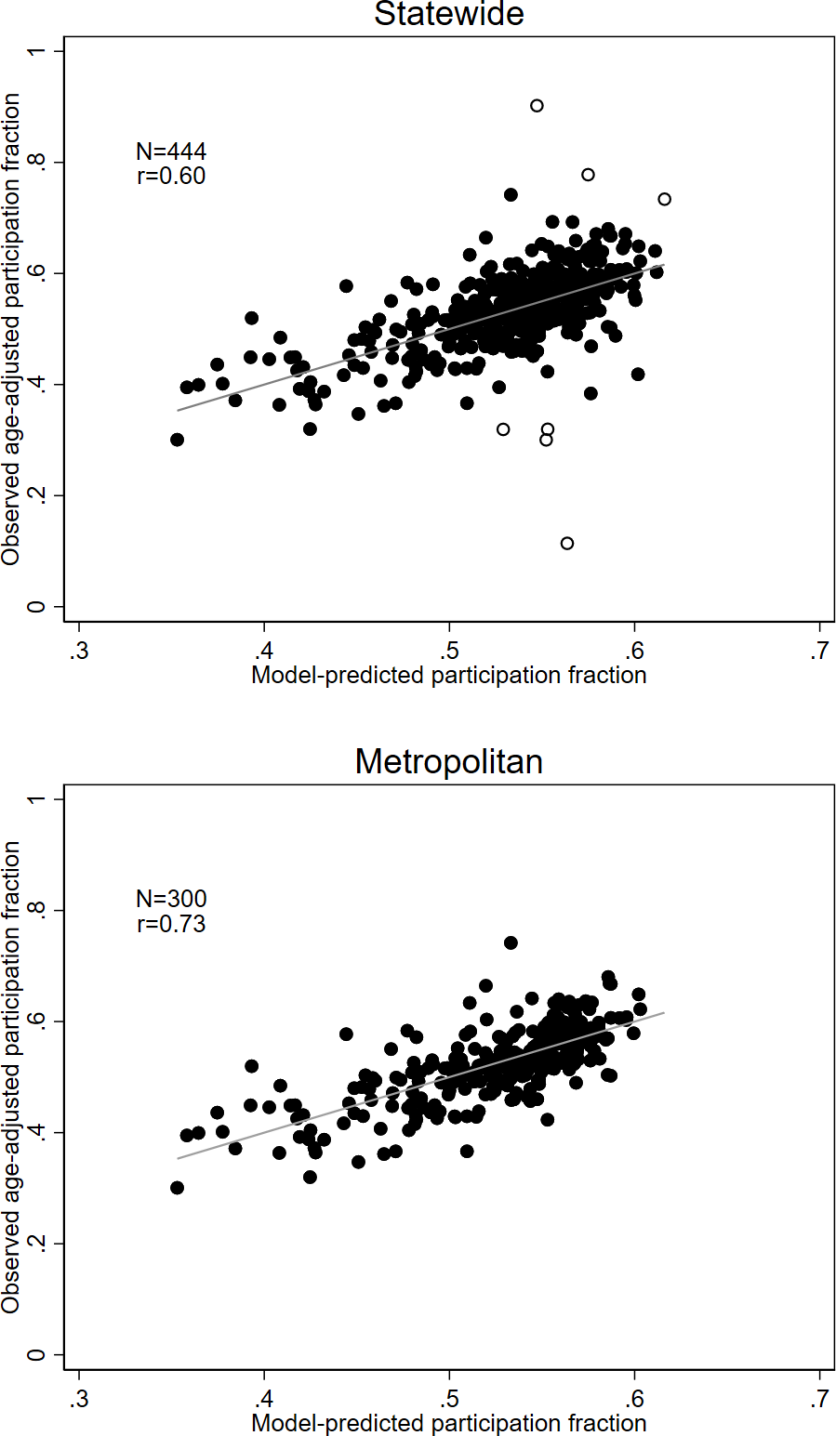
Relationship between observed screening participation and those predicted from a regression model using all nine socio-economic and ethnocultural measures, for the entire state (top panel) and metropolitan areas only (bottom panel). Identified outliers (top panel) are represented by open circles.

**Figure 1** illustrates the relationship between observed screening participation by area and that predicted by the model that included all nine separate socio-economic and ethnocultural measures. This graph (top panel) shows that there were some outlying values that are not well fitted by the regression model. Some of these outlying values may be explained by factors specific to certain areas (open circles). Some outliers representing higher levels of screening are due to some specific initiatives to improve screening participation in a rural region of central Victoria. Some areas with lower than expected participation were near the state border, where a nearby breast screening clinic is in the neighbouring state, which does not report mammographic screening to BreastScreen Victoria. These areas are adjacent to areas which were excluded from the dataset for that reason. There were no similar outliers in metropolitan areas (bottom panel). In metropolitan areas, more than half the variance (r^2^=.53; r=.73) in screening participation by area was accounted for by the socio-economic and ethnocultural characteristics of those areas.

## Discussion

Our area-based analysis of disparities in breast screening participation revealed a high degree of congruence between the socio-economic and ethnocultural characteristics of statistical areas, as they relate to breast screening. Notwithstanding this strong relationship, we found that ethnocultural factors taken together could account for a greater proportion of the variation in area-based screening participation than socio-economic factors. Notably, our area-based measures are consistent with the recent findings of the Australian Institute of Health and Welfare (17)) regarding language spoken at home. However, it is important to note that conclusions drawn from a particular set of measures are limited by the extent to which those measures fully capture the domain of interest. It is possible that the ethnocultural measures used in this study better capture the relevant variation between areas than do the socio-economic measures.

One limitation of this study is that estimated screening participation is subject to some known biases. An unknown proportion of women were non-citizens (not listed on the electoral roll) and therefore were not invited to free screening. These women are included in Census estimates of area population. Part of the reason that areas with higher proportions of migrants have lower screening participation may be due to this factor.

A further limitation is that some women would have been screened at a private clinic and were not included in the BreastScreen data. It is difficult to estimate the proportion of women screened privately using available data. Jacklyn et al. (2017) were able to access data from Australia’s Medicare Benefits Scheme regarding the proportion of women in the age groups eligible for screening who made claims for bilateral mammography in the state of New South Wales (3) (privately insured patients are eligible for a partial refund from the Medicare scheme). From 1996, when the free BreastScreen program was fully rolled out, approximately 7% of women within screening age range accessed bilateral mammography. While it is not specified in the Medicare records whether these mammograms were for screening or diagnostic purposes, Jacklyn et al. (2017) estimated that at least half, possibly more, were for screening (3). The use of private screening services may partly explain some of the non-linear relationships between individual measures of higher SES and screening participation, whereby the most advantaged and most disadvantaged women have lower screening participation than those in the middle of the range. It may be reasonably assumed that women with higher SES are more likely to use private screening services for reasons of convenience, given that the publicly funded program is free of cost. In Australia, less than 35% of those in the lowest quintile of the SEIFA index of socio-economic disadvantage have private health insurance, compared to nearly 80% of those in the highest quintile (23).

Another limitation of the present study is that it is area-based. Therefore, and in common with all studies that use area-based measures, inferences are potentially subject to the ecological fallacy (24). It is not possible to directly link, for example, the proportion of persons of a certain religion or country of birth in a given area to an individual’s screening participation. There can be strong relationships between a feature with a small number of people in the category and screening participation, but there may not be enough individuals in these categories to account for the observed variation in screening participation: the feature may rather be a marker for some other causally related characteristic of the area. Nonetheless, the relationships uncovered in this study were strong, considering their indirect nature.

Strengths of the present study include the large sample, covering the state of Victoria, the broad range of measures investigated, and the precautions taken to avoid overfitting in the analysis, given the large number of categories available in each of the area-based measures.

The use of separate socio-economic indices uncovered a much stronger gradient in breast screening participation compared to composite SEIFA indices, especially SEIFA disadvantage (IRSD) alone. This confirms the cautionary advice of McKracken (2001), who pointed out that the use of a SEIFA composite score may only capture around a third of the information represented by a set of statistical measures (20). Notably, in the present study IRSD alone accounted for only a small proportion of variance in screening participation, consistent with the findings of a recent national study using that measure (7). The use of separate measures, within a multiple regression framework, revealed a stronger relationship. Including an area-based measure of remoteness from major centres did not improve the fit of models that included separate measures of socio-economic and ethnocultural characteristics. This suggests that distance from major cities may not be a barrier to screening. The BreastScreen Victoria program includes a number of rural screening clinics as well as a mobile mammography service (25).

The association between ethnocultural background and SES is complex. ABS Census data shows that, on average, while Arab-Australians have lower SES than Australian-born residents, Chinese-Australians have the one of the highest average levels of SES among major migrant groups — higher than the Australian born — with Indian-Australians having comparable levels of SES to the general community (19). Yet all these migrant groups have lower mammographic screening participation compared to the Australian-born population in samples of convenience (12–14). That suggests that cultural and linguistic barriers may be driving the disparity in screening participation between these ethnocultural groups and the Australian-born.

Given that in Australia there is no direct cost associated with mammographic screening through BreastScreen, in principle, economic disadvantage should not present a barrier to screening. In the general population, it has been shown that knowledge and beliefs are stronger determinants of decisions to participate in breast screening than SES (15). It is possible that knowledge and beliefs act as mediators between SES and screening behaviour, that is, differing levels of knowledge and/or beliefs are associated with differing levels of SES. Alternatively, they may act independently of SES in determining screening behaviour.

Our findings highlight the need to consider ethnocultural factors as confounders of socio-economic factors. They support the strategy of targeting migrant groups to reduce disparities in screening participation (26) and exploring the reasons, cultural or otherwise, for lower participation in screening within those groups (9).

## Data availability statement

The data analyzed in this study is subject to the following licenses/restrictions: The breast cancer screening data is confidential and subject to privacy laws. It can be obtained on request from Breast Screen Victoria but not publicly disseminated. Requests to access these datasets should be directed to gwstuart@unimelb.edu.

## Ethics statement

The studies involving human participants were reviewed and approved by Cancer Council Victoria Human Ethics Committee. Written informed consent for participation was not required for this study in accordance with the national legislation and the institutional requirements.

## Author contributions

GS: Conceptualisation; Methodology, Formal analysis, Data curation, Writing – original draft, Writing – Review and editing. JC: Conceptualisation, Formal analysis, Writing – Review and editing. RM: Conceptualisation, Writing – Review and Editing, Supervision. All authors contributed to the article and approved the submitted version

## Funding

This work was supported by an NHMRC Partnership Grant (number 1150012).

## Acknowledgments

The authors thank BreastScreen Victoria for making data available for analysis and Julie Bassett for helpful comments on an earlier version of the manuscript

## Conflict of interest

The authors declare that the research was conducted in the absence of any commercial or financial relationships that could be construed as a potential conflict of interest.

## Publisher’s note

All claims expressed in this article are solely those of the authors and do not necessarily represent those of their affiliated organizations, or those of the publisher, the editors and the reviewers. Any product that may be evaluated in this article, or claim that may be made by its manufacturer, is not guaranteed or endorsed by the publisher.
