# Supplementary material for: Socio-economic and ethnocultural influences on geographical disparities in breast cancer screening participation in Victoria, Australia
Source: Front Oncol. 2022 Nov 29;12:980879. doi: 10.3389/fonc.2022.980879 (PMC9745803; doi:10.3389/fonc.2022.980879)
Supplement: Supplementary file 1 [file DataSheet_1.docx]

**Supplementary Material**

This document provides details of the construction of individual scores representing the association between area-based measures of socio-economic status and ethnocultural diversity and breast screening participation. The general approach is described in the main body of the paper. The sections below provide specific details of each score.

In the tables below, the first column represents the univariate correlation between the proportion of people in each individual category within each area and age-adjusted screening participation with the unit of analysis being statistical area. All associations were checked for nonlinearity using scatterplots. For example, the graph below shows the relationship between average weekly income in each statistical area (averaged across income bands) and screening participation.


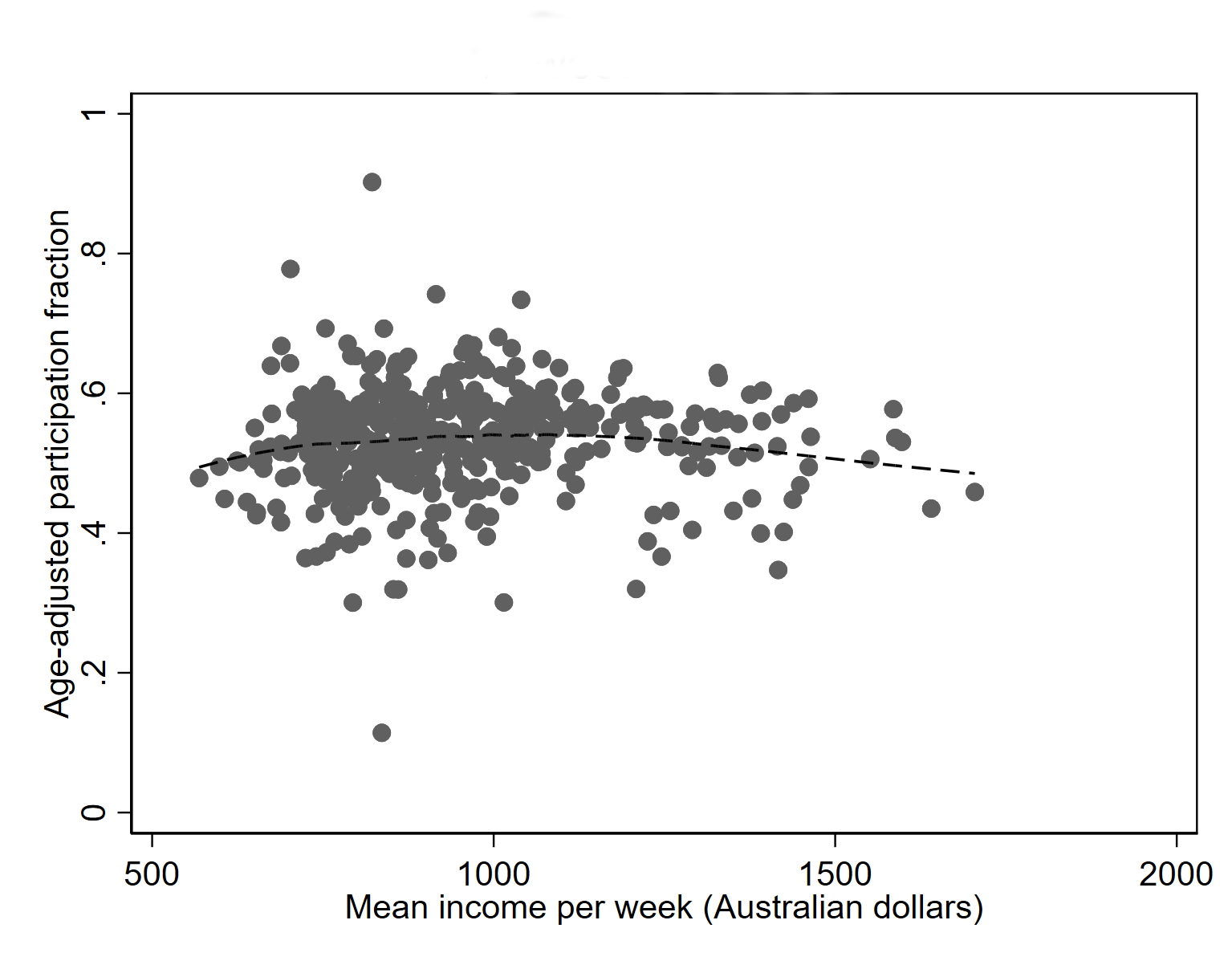


Figure S1: Relationship between income in statistical areas and screening participation. The dashed line represents a Lowess smoother.

The scatterplot indicates that there might be a quadratic relationship between income and screening participation. Quadratic terms were derived from squared terms for an independent variable, statistically conditioned on linear terms. This yields an orthogonal (additive) estimate of the quadratic term. This term is then used in a regression, and if it significantly predicted the outcome it was included in univariate and multivariate regression models. In this case the quadratic term is significant (r=-.151, F[1,442] = 10.29, p=.001). The negative sign of the correlation indicates an inverted U-shaped relationship, as noted under the tables where appropriate. There were no significant cubic or higher polynomial relationships observed during screening for nonlinear relationships.

The third and fourth columns in each table represent the squared univariate correlation (proportion of variance in screening explained) and the significance of the univariate relationships. The fifth and sixth columns show semi-partial correlations and statistical significance for reduced models. These reduced models were derived by dropping terms from regression models, removing the term with the smallest semi-partial correlation at each step until the model fit was significantly worse than the starting model, and then restoring the last term. The fit was considered significantly worse if a $\chi^{2}$test on the terms dropped from the starting model (df=number of terms dropped from the starting model) had p < 0.05 as shown below the table. This procedure was designed to avoid overfitting.

In the context of spatial regression we continued with a procedure to prune non-significant terms from the set of terms retained using standard regression. The final spatial models could have fewer regression terms than standard regressions if dropping some terms did not significantly reduce the fit of spatial regression models. At each step the term with the least significant Z-score was dropped if it had p > 0.05. The fit of the reduced spatial mode was compared with the spatial model including all terms from the final standard regression model using a $\chi^{2}$test with degrees of freedom corresponding to the number of terms dropped. If the fit was significantly worse that term was restored to give the final model. Given there is no equivalent of semi-partial correlation in spatial regression, Z-values and p-values for individual spatial regression coefficients are presented in the last two columns of the table.

Below each table are shown the R^2^ and adjusted R^2^ values for the full model with all univariate parameters, and the reduced model after some terms were dropped, and the likelihood ratio $\chi^{2}$ test comparing the fit of the two conventional regression models. Below that are shown the likelihood ratio $\chi^{2}$test of the fit of the reduced spatial model, relative to the starting model derived from standard regression, and the $\chi^{2}$ test of spatial autocorrelation in the residuals. When this autocorrelation term was included, other spatial autocorrelation terms for independent variables or for the dependent variable itself were generally not significant, or exhibited only borderline significance, and there was no meaningful improvement in model fit. Consequently, in the final models we used the autocorrelation term for the residuals but no autocorrelation terms for the dependent or independent variables. The fitted (predicted) values from the final regression models were then retained as the summary scores for that measure.

Table S1. Birthplace

| Region | Univariate r | Univariate r^2^ | p-value | Semi-partial r^2^ | p-value | Spatial Z | Spatial p-value |
| --- | --- | --- | --- | --- | --- | --- | --- |
| Australia | .4166 | .174 | <.0001 | .173 | <.0001 | 8.55 | <.001 |
| New Zealand | -.3941 | .155 | <.0001 | — | — | — | — |
| Polynesia | -.2757 | .076 | <.0001 | — | — | — | — |
| U.K. (excl. Ireland) | .0793 | .006 | 0.0953 | .009 | .016 | — | — |
| Western Europe | .0141 | .0002 | 0.7664 | — | — | — | — |
| Northern Europe | -.1473 | .022 | 0.0019 | — | — | — | — |
| S Europe | .0710 | .005 | 0.1352 | .018 | .001 | 2.77 | .006 |
| SE Europe | -.1757 | .031 | 0.0012 | .030 | <.0001 | 2.11 | .035 |
| E Europe | -.0893 | .008 | 0.0602 | .009 | <.0001 | 3.35 | .001 |
| N Africa | -.2641 | .070 | <.0001 | .008 | .028 | — | — |
| Middle East | -.2021 | .041 | <.0001 | .019 | .001 | 2.03 | .043 |
| Mainland SE Asia | -.1237 | .015 | 0.0091 | .085 | <.0001 | 5.67 | <.001 |
| Maritime SE Asia | -.2959 | .088 | <.0001 | — | — | — | — |
| Chinese Asia | -.1439 | .021 | 0.0024 | .097 | <.0001 | 5.22 | <.001 |
| Japan/Korea | -.2140 | .046 | <.0001 | — | — | — | — |
| Southern Asia | -.3413 | .116 | <.0001 | .033 | <.0001 | 3.33 | .001 |
| N America | -.1354 | .018 | 0.0042 | — | — | — | — |
| S America | -.3417 | .117 | <.0001 | — | — | — | — |
| S and E Africa | -.2615 | .068 | <.0001 | — | — | — | — |

Full model: Overall R^2^=.338, Adjusted R^2^=.3082

Reduced model: Overall R^2^=.317, Adjusted R^2^=.302.

Model Comparison: $\chi^{2}(9)$=13.47, p=.143.

Reduced spatial model: $\chi^{2}(2)$=4.82, p=.080.

Residual autocorrelation term:$\chi^{2}(1)$=16.68, p <.0001

Note: Included in standard models were three regions with non-significant univariate effects but significant multivariate suppressor effects (UK, S. Europe and E. Europe).

Table S2. English language ability

The categories for proficiency in spoken English, for speakers of other languages, are “not at all”, “not well”, and “very well”. A fourth category represents the proportion of respondent who speak English only. These categories are weighted, in order, with equal weights for “very well” and for monolingual English speakers to produce a continuous score for English language proficiency for each area.

| English Score | Univariate r | Univariate r^2^ | p-value | Semi-partial r^2^ | p-value | Spatial Z | Spatial p-value |
| --- | --- | --- | --- | --- | --- | --- | --- |
| English | .3174 | .101 | <.0001 | .101 | <.0001 | 5.61 | <.001 |
| English^2^ | .0681 | .005 | .163 | — | — | — | — |

Positive quadratic: $\cup$ Negative quadratic: $\cap$

Full model: Overall R^2^=.106, Adjusted R^2^= .101

Reduced Model: Overall R^2^=.101, Adjusted R^2^=.099. Sum of semi-partials = .101

Model Comparison: $\chi^{2}(1)$=2.47, p=.116.

Reduced spatial model: Not applicable

Residual autocorrelation term: $\chi^{2}(1)$=75.18, p <.0001

Table S3. Religion

| Religion | Univariate r | Univariate r^2^ | p-value | Semi-partial r^2^ | p-value | Spatial Z | Spatial p-value |
| --- | --- | --- | --- | --- | --- | --- | --- |
| Buddhism | -.1825 | .033 | .0001 | — | — | — | — |
| Buddhism^2^ | .2481 | .062 | <.0001 | .015 | .003 | 2.99 | .003 |
| Christianity | .3791 | .144 | <.0001 | — | — | — | — |
| Hinduism | -.3381 | .114 | <.0001 | .013 | .006 | -2.50 | .013 |
| Islam | -.2975 | .089 | <.0001 | .024 | .0002 | -3.31 | .001 |
| Islam^2^ | .1898 | .036 | .0001 | — | — | — | — |
| Other | -.3084 | .095 | <.0001 | .019 | .0006 | -2.58 | .010 |
| Secular | .0888 | .008 | .0614 | .013 | .005 | -2.54 | .011 |
| Secular^2^ | -.2829 | .080 | <.0001 | — | — | — | — |
| Not Stated | -.2581 | .067 | <.0001 | .097 | <.0001 | -6.53 | <.001 |
| Not Stated^2^ | .0944 | .009 | .047 | .0125 | .006 | 2.07 | .038 |

Positive quadratic: $\cup$ Negative quadratic: $\cap$

Full model: Overall R^2^=.308, Adjusted R^2^= .292

Reduced Model: Overall R^2^=.298, Adjusted R^2^=.287.

Model Comparison: $\chi^{2}(4)$=6.23, p=.183.

Reduced spatial model: Not applicable

Residual autocorrelation term: $\chi^{2}(1)$=17.93, p <.0001

Table S4. Language Spoken at Home

| Language Spoken at Home | Univariate r | Univariate r^2^ | p-value | Semi-partial r^2^ | p-value | Spatial Z | Spatial p-value |
| --- | --- | --- | --- | --- | --- | --- | --- |
| N. European* | .3659 | .134 | <.0001 | .124 | <.0001 | 6.76 | <.001 |
| S. European | -.1226 | .015 | .001 | .069 | <.0001 | 5.05 | <.001 |
| E. European | -.1805 | .033 | .0001 | .016 | .002 | 2.10 | .035 |
| SW Central Asian | -.2230 | .050 | <.0001 | .057 | <.0001 | 4.12 | <.001 |
| S Asian | -.3394 | .115 | <.0001 | .024 | .0002 | 2.71 | .007 |
| SE Asian | -.1765 | .031 | .0002 | .080 | <.0001 | 5.41 | <.001 |
| E Asian | -.1360 | .018 | .0004 | .075 | <.0001 | 4.33 | <.001 |
| Other | -.3579 | .128 | <.0001 | — | — | — | — |
| Other^2^ | .1362 | .019 | .0040 | — | — | — | — |
| Supplementary | -.3530 | .124 | <.0001 | — | — | — | — |

Positive quadratic: $\cup$ Negative quadratic: $\cap$

*Mainly English

Full model: Overall R^2^=.266, Adjusted R^2^=.250

Reduced Model: Overall R^2^=.250, Adjusted R^2^=.237.

Model Comparison: $\chi^{2}(3)$=10.13, p=.017.

Reduced spatial model: Not applicable

Residual autocorrelation term: $\chi^{2}(1)$=33.19, p <.0001

Note: There was a slight but significant reduction in fit when “other” and “supplementary” were not included (1.2% for adjusted R^2^), but the model is more interpretable without them.

Table S5. Recency of migration

| Migration measure | Univariate r | Univariate r^2^ | p-value | Semi-partial r^2^ | p-value | Spatial Z | Spatial p-value |
| --- | --- | --- | --- | --- | --- | --- | --- |
| Migrant proportion | -.362 | .131 | <.0001 | .099 | .0001 | -5.76 | <.001 |
| Recency of migration | .277 | .077 | <.0001 | — | — | — | — |
| Recency^2^ | -.342 | .117 | <.0001 | .085 | <.0001 | -4.84 | <.001 |
| Proportion*  Recency | .304 | .093 | <.0001 | .008 | .034 | — | — |
| Proportion*  Recency^2^ | -.068 | .005 | .153 | — | — | — | — |

Positive quadratic: $\cup$ Negative quadratic: $\cap$

Full model: Overall R^2^=.228, Adjusted R^2^=.219

Reduced Model: Overall R^2^=.223, Adjusted R^2^=.222.

Model Comparison: $\chi^{2}\left( 2 \right)=$ 0.74, p=.692

Reduced spatial model: $\chi^{2}\left( 1 \right)=$ 3.76, p=.053

Residual autocorrelation term: $\chi^{2}(1)$=38.00, p <.0001

Note: Product terms for both quadratic effects and interactions are orthogonalised against lower-order terms.

Table S6. Income

| Income measure | Univariate r | Univariate r^2^ | p-value | Semi-partial r^2^ | p-value | Spatial Z | Spatial p-value |
| --- | --- | --- | --- | --- | --- | --- | --- |
| Mean Income | .0201 | .0004 | .673 | — | — | — | — |
| Mean Income^2^ | -.1510 | .023 | .001 | .009 | .03 | — | — |
| Nil Income | -.2318 | .054 | <.0001 | .102 | <.0001 | -5.10 | <.001 |
| Negative Income | -.2134 | .046 | <.0001 | — | — | — | — |
| Not stated | -.2162 | .047 | <.0001 | .072 | <.0001 | -5.26 | <.001 |

Positive quadratic: $\cup$ Negative quadratic: $\cap$

Full model: Overall R^2^=.160, Adjusted R^2^=.150

Reduced Model: Overall R^2^=.144, Adjusted R^2^=138.

Model Comparison: $\chi^{2}\left( 2 \right)=$2.52, p=.283

Reduced spatial model: $\chi^{2}\left( 2 \right)=$4.73, p=.094

Residual autocorrelation term: $\chi^{2}(1)$=55.28, p <.0001

Table S7. Education

| Education Measure | Univariate r | Univariate r^2^ | p-value | Semi-partial r^2^ | p-value | Spatial Z | Spatial p-value |
| --- | --- | --- | --- | --- | --- | --- | --- |
| Postgraduate | -.1707 | .029 | .0003 | .070 | <.0001 | -4.97 | <.001 |
| Graduate Dip. | .1064 | .011 | .025 | .093 | <.0001 | 6.39 | <.001 |
| Bachelor^2^ | -.1049 | .011 | .027 | .008 | .031 | — | — |
| Certificate4 | .2015 | .041 | <.0001 | — | — | — | — |
| Secondary Yr 10 | .1006 | .010 | .034 | — | — | — | — |
| Secondary Yr 10^2^ | -.2276 | .052 | <.0001 | .014 | .005 | — | — |
| Secondary Yr 9 | .1172 | .014 | .014 | .015 | .004 | 2.39 | .017 |
| Secondary Yr 9^2^ | -.1443 | .021 | .002 | — | — | — | — |
| Not Stated | -.1751 | .031 | .0002 | .057 | <.0001 | -4.23 | <.001 |

Positive quadratic: $\cup$ Negative quadratic: $\cap$

Full model: Overall R^2^=.242, Adjusted R^2^=.226

Reduced Model: Overall R^2^=.225, Adjusted R^2^=.216.

Model Comparison: $\chi^{2}\left( 3 \right)=$5.04, p=.169

Reduced spatial model: $\chi^{2}\left( 5 \right)=$6.89, p=.229

Residual autocorrelation term: $\chi^{2}(1)$=39.13, p <.0001

Table S8. Occupation

|  | Univariate r | Univariate r^2^ | p-value | Semi-partial r^2^ | p-value | Spatial Z | Spatial p-value |
| --- | --- | --- | --- | --- | --- | --- | --- |
| Managers | .1098 | .012 | .021 | .0233 | .0006 | — | — |
| Managers^2^ | -.1653 | .027 | .0005 | — | — | — | — |
| Professionals^2^ | -.2120 | .045 | <.0001 | — | — | — | — |
| Clerical/Admin | .1014 | .010 | .033 | — | — | — | — |
| Sales | .1885 | .036 | .0001 | .0617 | <.0001 | 3.97 | <.001 |
| Operators/Drivers | -.1891 | .036 | .0001 | .0147 | .006 | -3.65 | <.001 |
| Operators/Drivers^2^ | -.2165 | .047 | <.0001 | .0253 | .0004 | — | — |
| Not Stated | -.1704 | .029 | .0003 | — | — | — | — |

Positive quadratic: $\cup$ Negative quadratic: $\cap$

Full model: Overall R^2^=.148, Adjusted R^2^=132.

Reduced Model: Overall R^2^=.141, Adjusted R^2^=.136.

Model Comparison: $\chi^{2}\left( 4 \right)=$2.04, p=.728

Reduced spatial model: $\chi^{2}\left( 2 \right)=$4.29, p=.117

Residual autocorrelation term: $\chi^{2}(1)$=80.14, p <.0001

Table S9. Employment

| Employment Category | Univariate r | Univariate r^2^ | p-value | Semi-partial r^2^ | p-value | Spatial Z | Spatial p-value |
| --- | --- | --- | --- | --- | --- | --- | --- |
| Employed FT^2^ | -.2401 | .058 | <.0001 | — | — | — | — |
| Employed PT | .3540 | .125 | <.0001 | .023 | .0003 | 3.69 | <.001 |
| Unemployed FT | -.3180 | .101 | <.0001 | — | — | — | — |
| Unemployed PT* | -.2655 | .070 | <.0001 | .065 | <.0001 | -4.68 | <.001 |
| Not In Workforce^2^ | -.2167 | .047 | <.0001 | .012 | .012 | — | — |
| Not Stated | -.2779 | .077 | <.0001 | .024 | .0003 | -3.08 | .002 |

Positive quadratic: $\cup$ Negative quadratic: $\cap$

*Seeking part-time employment

Full model: Overall R^2^=.219, Adjusted R^2^=.208

Reduced Model: Overall R^2^=.213, Adjusted R^2^=.206.

Reduced spatial model: $\chi^{2}\left( 1 \right)=$2.16, p=.141

Residual autocorrelation term: $\chi^{2}(1)$=47.17, p <.0001
